# Phylogeny and Evolutionary Patterns in the Dwarf Crayfish Subfamily (Decapoda: Cambarellinae)

**DOI:** 10.1371/journal.pone.0048233

**Published:** 2012-11-14

**Authors:** Carlos Pedraza-Lara, Ignacio Doadrio, Jesse W. Breinholt, Keith A. Crandall

**Affiliations:** 1 Departamento de Biodiversidad y Biología Evolutiva, Museo Nacional de Ciencias Naturales, CSIC, Madrid, Spain; 2 Instituto de Biología, Universidad Nacional Autónoma de México, Distrito Federal, México; 3 Department of Biology, Brigham Young University, Provo, Utah, United States of America; 4 Computational Biology Institute, George Washington University, Ashburn, Virginia, United States of America; Institute of Evolutionary Biology (CSIC-UPF), Spain

## Abstract

The Dwarf crayfish or Cambarellinae, is a morphologically singular subfamily of decapod crustaceans that contains only one genus, *Cambarellus*. Its intriguing distribution, along the river basins of the Gulf Coast of United States (Gulf Group) and into Central México (Mexican Group), has until now lacked of satisfactory explanation. This study provides a comprehensive sampling of most of the extant species of *Cambarellus* and sheds light on its evolutionary history, systematics and biogeography. We tested the impact of Gulf Group versus Mexican Group geography on rates of cladogenesis using a maximum likelihood framework, testing different models of birth/extinction of lineages. We propose a comprehensive phylogenetic hypothesis for the subfamily based on mitochondrial and nuclear loci (3,833 bp) using Bayesian and Maximum Likelihood methods. The phylogenetic structure found two phylogenetic groups associated to the two main geographic components (Gulf Group and Mexican Group) and is partially consistent with the historical structure of river basins. The previous hypothesis, which divided the genus into three subgenera based on genitalia morphology was only partially supported (P = 0.047), resulting in a paraphyletic subgenus *Pandicambarus*. We found at least two cases in which phylogenetic structure failed to recover monophyly of recognized species while detecting several cases of cryptic diversity, corresponding to lineages not assigned to any described species. Cladogenetic patterns in the entire subfamily are better explained by an allopatric model of speciation. Diversification analyses showed similar cladogenesis patterns between both groups and did not significantly differ from the constant rate models. While cladogenesis in the Gulf Group is coincident in time with changes in the sea levels, in the Mexican Group, cladogenesis is congruent with the formation of the Trans-Mexican Volcanic Belt. Our results show how similar allopatric divergence in freshwater organisms can be promoted through diverse vicariant factors.

## Introduction

**Figure 1:**
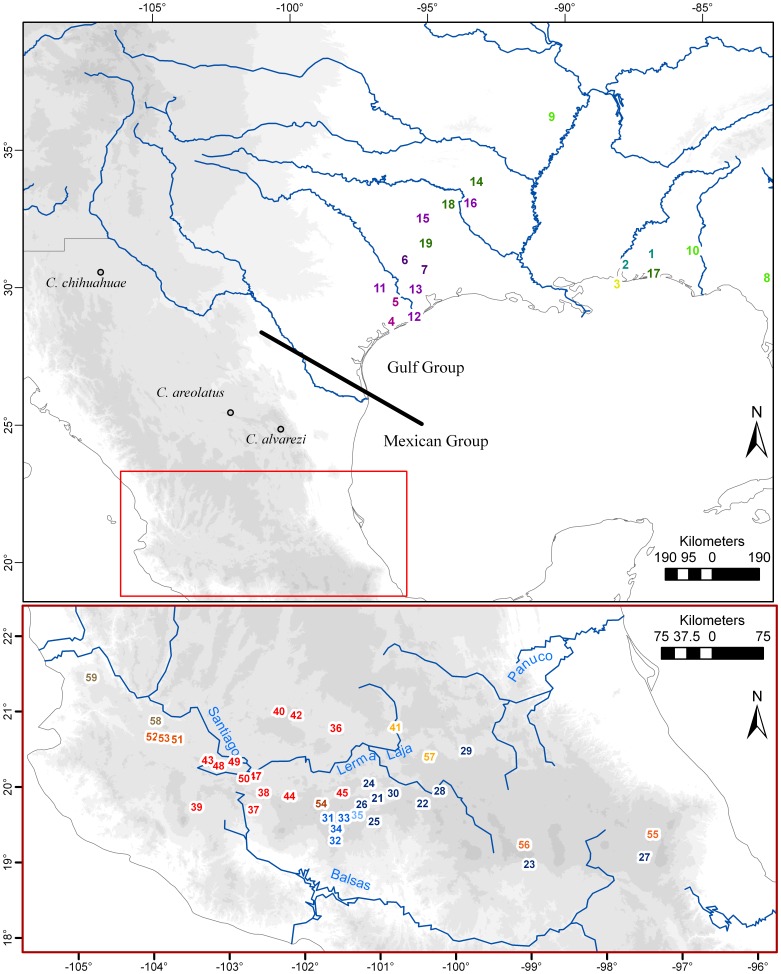
Map of localities sampled. Map of localities sampled in this study, numbers are referred to in Table 1. Sample locations are colored to represent different clades recovered by phylogenetic analyses (see Fig. 3). Open circles correspond to the only locality records for the three species not included in the analyses as they were not found during sampling, or did not amplify during PCR reactions. Gray background refers to elevation (500–6000 m).

The freshwater crayfish subfamily Cambarellinae is comprised of the unique genus *Cambarellus*, with 17 recognized species and a disjunctive distribution across the freshwater streams of the Gulf Cost of the United States and North and Central México (Fig. 1) [1]. The subfamily is unique because of the exceptionally small body size of its species. They typically reach only 4 cm compared to most crayfish averaging a maximum body size of >5 cm; hence, the reference to the genus as the “Dwarf” crayfishes. Their distribution goes from the Swanee River in northern Florida, eastward through the southern Mississippi River watershed to southern Illinois and continues southwest to the Nueces River in Texas [2], [3]. In México, *Cambarellus* has a discontinuous distribution with three distant and isolated populations from the northern states of Chihuahua, Coahuila and Nuevo León and then along the Trans-Mexican Volcanic Belt (TMVB) [4], [5]. The genus contains species largely inhabiting lakes and lentic habitats. The evolutionary history of such a broad and disjunct distribution of species is unclear and our goal with this study is to shed some light on the biogeography of the Cambarellinae.

**Table 1:** Sampling localities and Genbank accession numbers from individuals of *Cambarellus* used in this study.

+	Species id fromthis study	Subgenus(Fitzpatrick,1983)	GeneBank accession numbers				
			16S	12S	cox1	28S	H3
1	*Cambarellus blacki*	*Pandicambarus*	JX127836	JX127697	JX127977	JX127568	JX127429
1	*Cambarellus blacki*	*Pandicambarus*	JX127837	JX127698	JX127978	JX127569	JX127430
2	*Cambarellus diminutus* *	*Pandicambarus*	JX127810		JX127953	JX127545	JX127405
3	*Cambarellus lesliei* *	*Pandicambarus*	JX127809		JX127952	JX127544	JX127404
4	*Cambarellus ninae*	*Pandicambarus*	JX127814		JX127957	JX127549	JX127409
5	*Cambarellus ninae* **	*Pandicambarus*	JX127833	JX127694	JX127974	JX127565	JX127426
6	*Cambarellus puer1233*	*Pandicambarus*	JX127822	JX127686	JX127965	JX127557	JX127417
7	*Cambarellus puer*	*Pandicambarus*	JX127815		JX127958	JX127550	JX127410
8	*Cambarellus schmitti*	*Pandicambarus*	JX127811		JX127954	JX127546	JX127406
9	*Cambarellus schmitti*	*Pandicambarus*	JX127838-	JX127699-	JX127979-	JX127570-	JX127431-
			JX127855	JX127714	JX127996	JX127587	JX127447
10	*Cambarellus schmitti*	*Pandicambarus*	JX127856	JX127715	JX127997		JX127448
11	*Cambarellus texanus*	*Pandicambarus*	JX127832				
12	*Cambarellus texanus* ***	*Pandicambarus*	JX127834	JX127695	JX127975	JX127566	JX127427
13	*Cambarellus texanus*	*Pandicambarus*	JX127819 -	JX127683 –	JX127962 –	JX127554 –	JX127414 –
			JX127821	JX127685	JX127964	JX127556	JX127416
14	*Cambarellus shufeldtii*	*Dirigicambarus*	JX127812		JX127955	JX127547	JX127407
15	*Cambarellus shufeldtii*	*Dirigicambarus*	JX127816		JX127959	JX127551	JX127411
16	*Cambarellus shufeldtii*	*Dirigicambarus*	JX127817		JX127960	JX127552	JX127412
17	*Cambarellus shufeldtii*	*Dirigicambarus*					
18	*Cambarellus shufeldtii*	*Dirigicambarus*	JX127835	JX127696	JX127976	JX127567	JX127428
19	*Cambarellus shufeldtii*	*Dirigicambarus*	JX127857		JX127998	JX127588	JX127449
20	*Cambarellus shufeldtii*	*Dirigicambarus*	JX127818		JX127961	JX127553	JX127413
21	*Cambarellus zempoalensis*	*Cambarellus*	JX127725	JX127599	JX127868	JX127460	JX127320
21	*Cambarellus zempoalensis*	*Cambarellus*	JX127770	JX127644	JX127913	JX127505	JX127365
22	*Cambarellus zempoalensis*	*Cambarellus*	JX127747	JX127621	JX127890	JX127482	JX127342
22	*Cambarellus zempoalensis*	*Cambarellus*	JX127759	JX127633	JX127902	JX12749	JX127354
22	*Cambarellus zempoalensis*	*Cambarellus*	JX127772	JX127646	JX127915	JX127507	JX127367
22	*Cambarellus zempoalensis*	*Cambarellus*	JX127773	JX127647	JX127916	JX127508	JX127368
23	*Cambarellus zempoalensis*	*Cambarellus*	JX127750	JX127624	JX127893	JX127485	JX127345
23	*Cambarellus zempoalensis*	*Cambarellus*	JX127756	JX127630	JX127899	JX127491	JX127351
24	*Cambarellus zempoalensis*	*Cambarellus*	JX127786	JX127660	JX127929	JX127521	JX127381
25	*Cambarellus zempoalensis*	*Cambarellus*	JX127744	JX127618	JX127887	JX127479,	JX127339
25	*Cambarellus zempoalensis*	*Cambarellus*	JX127753	JX127627	JX127896	JX127488	JX127348
25	*Cambarellus zempoalensis*	*Cambarellus*	JX127794	JX127668	JX127937	JX127529	JX127389
26	*Cambarellus zempoalensis*	*Cambarellus*	JX127743	JX127617	JX127886	JX127478	JX127338
26	*Cambarellus zempoalensis*	*Cambarellus*	JX127755	JX127629	JX127898	JX127490	JX127350
26	*Cambarellus zempoalensis*	*Cambarellus*	JX127765	JX127639	JX127908	JX127500	JX127360
26	*Cambarellus zempoalensis*	*Cambarellus*	JX127791	JX127665	JX127934	JX127526	JX127386
27	*Cambarellus zempoalensis*	*Cambarellus*	JX127732	JX127606	JX127875	JX127467	JX127327
28	*Cambarellus zempoalensis*	*Cambarellus*	JX127771	JX127645,	JX127914	JX127506	JX127366
28	*Cambarellus zempoalensis*	*Cambarellus*	JX127793	JX127667	JX127936	JX127528	JX127388
29	*Cambarellus zempoalensis*	*Cambarellus*	JX127736	JX127610	JX127879	JX127471	JX127331
29	*Cambarellus zempoalensis*	*Cambarellus*	JX127754	JX127628	JX127897	JX127489	JX127349
29	*Cambarellus zempoalensis*	*Cambarellus*	JX127789	JX127663	JX127932	JX127524	JX127384
29	*Cambarellus zempoalensis*	*Cambarellus*	JX127805	JX127679	JX127948	JX127540	JX127400
30	*Cambarellus zempoalensis*	*Cambarellus*	JX127798	JX127672	JX127941	JX127533	JX127393
30	*Cambarellus zempoalensis*	*Cambarellus*	JX127799	JX127673	JX127942	JX127534	JX127394
31	*Cambarellus patzcuarensis*	*Cambarellus*	JX127728	JX127602	JX127871	JX127463	JX127323
31	*Cambarellus patzcuarensis*	*Cambarellus*	JX127740	JX127614	JX127883	JX127475	JX127335
31	*Cambarellus patzcuarensis*	*Cambarellus*	JX127751	JX127625	JX127894	JX127486	JX127346
31	*Cambarellus patzcuarensis*	*Cambarellus*	JX127774	JX127648	JX127917	JX127509	JX127369
32	*Cambarellus patzcuarensis*	*Cambarellus*	JX127802	JX127676	JX127945	JX127537	JX127397
32	*Cambarellus patzcuarensis*	*Cambarellus*	JX127803	JX127677	JX127946	JX127538	JX127398
33	*Cambarellus patzcuarensis*	*Cambarellus*	JX127741	JX127615	JX127884	JX127476	JX127336
33	*Cambarellus patzcuarensis*	*Cambarellus*	JX127775	JX127649	JX127918	JX127510	JX127370
34	*Cambarellus patzcuarensis*	*Cambarellus*	JX127779	JX127653	JX127922	JX127514	JX127374
35	*Cambarellus sp.* (cladeIII)	*Cambarellus*	JX127738	JX127612	JX127881	JX127473	JX127333
35	*Cambarellus sp.* (cladeIII)	*Cambarellus*	JX127752	JX127626	JX127895	JX127487	JX127347
36	*Cambarellus chapalanus*	*Cambarellus*	JX127726	JX127600	JX127869	JX127461	JX127321
36	*Cambarellus chapalanus*	*Cambarellus*	JX127760	JX127634	JX127903	JX127495	JX127355
37	*Cambarellus chapalanus*	*Cambarellus*	JX127733	JX127607	JX127876	JX127468	JX127328
37	*Cambarellus chapalanus*	*Cambarellus*	JX127734	JX127608	JX127877	JX127469	JX127329
37	*Cambarellus chapalanus*	*Cambarellus*	JX127737	JX127611	JX127880	JX127472	JX127332
37	*Cambarellus chapalanus*	*Cambarellus*	JX127764	JX127638	JX127907	JX127499	JX127359
37	*Cambarellus chapalanus*	*Cambarellus*	JX127830	JX127693	JX127972	JX127564	JX127425
38	*Cambarellus chapalanus*	*Cambarellus*	JX127795	JX127669	JX127938	JX127530	JX127390
38	*Cambarellus chapalanus*	*Cambarellus*	JX127796	JX127670	JX127939	JX127531	JX127391
38	*Cambarellus chapalanus*	*Cambarellus*	JX127800	JX127674	JX127943	JX127535	JX127395
39	*Cambarellus chapalanus*	*Cambarellus*	JX127742	JX127616	JX127885	JX127477	JX127337
39	*Cambarellus chapalanus*	*Cambarellus*	JX127766	JX127640	JX127909	JX127501	JX127361
40	*Cambarellus chapalanus*	*Cambarellus*	JX127783	JX127657	JX127926	JX127518	JX127378
41	*Cambarellus chapalanus*	*Cambarellus*	JX127748	JX127622	JX127891	JX127483	JX127343
41	*Cambarellus chapalanus*	*Cambarellus*	JX127749	JX127623	JX127892	JX127484	JX127344
41	*Cambarellus chapalanus*	*Cambarellus*	JX127768	JX127642	JX127911	JX127503	JX127363
41	*Cambarellus chapalanus*	*Cambarellus*	JX127792	JX127666	JX127935	JX127527	JX127387
41	*Cambarellus chapalanus*	*Cambarellus*	JX127797	JX127671	JX127940	JX127532	JX127392
42	*Cambarellus chapalanus*	*Cambarellus*	JX127781	JX127655	JX127924	JX127516	JX127376
43	*Cambarellus prolixus*	*Cambarellus*	JX127729	JX127603	JX127872	JX127464	JX127324
43	*Cambarellus prolixus*	*Cambarellus*	JX127761	JX127635	JX127904	JX127496	JX127356
43	*Cambarellus prolixus*	*Cambarellus*	JX127762	JX127636	JX127905	JX127497	JX127357
44	*Cambarellus chapalanus*	*Cambarellus*	JX127735	JX127609	JX127878	JX127470	JX127330
44	*Cambarellus chapalanus*	*Cambarellus*	JX127769	JX127643	JX127912	JX127504	JX127364
45	*Cambarellus chapalanus*	*Cambarellus*	JX127801	JX127675	JX127944	JX127536	JX127396
46	*Cambarellus chapalanus*	*Cambarellus*	JX127739	JX127613	JX127882	JX127474	JX127334
46	*Cambarellus chapalanus*	*Cambarellus*	JX127758	JX127632	JX127901	JX127493	JX127353
47	*Cambarellus chapalanus*	*Cambarellus*	JX127745	JX127619	JX127888	JX127480	JX127340
47	*Cambarellus chapalanus*	*Cambarellus*	JX127746	JX127620	JX127889	JX127481	JX127341
48	*Cambarellus prolixus*	*Cambarellus*	JX127806	JX127680	JX127949	JX127541	JX127401
48	*Cambarellus prolixus*	*Cambarellus*	JX127807	JX127681	JX127950	JX127542	JX127402
49	*Cambarellus prolixus*	*Cambarellus*	JX127808	JX127682	JX127951	JX127543	JX127403
50	*Cambarellus chapalanus*	*Cambarellus*	JX127804	JX127678	JX127947	JX127539	JX127399
51	*Cambarellus sp.* (clade V)	*Cambarellus*	JX127780	JX127654	JX127923	JX127515	JX127375
52	*Cambarellus sp. (*clade V)	*Cambarellus*	JX127782	JX127656	JX127925	JX127517	JX127377
53	*Cambarellus sp.* (clade V)	*Cambarellus*	JX127787	JX127661	JX127930	JX127522,	JX127382
53	*Cambarellus sp.* (clade V)	*Cambarellus*	JX127788	JX127662	JX127931	JX127523	JX127383
54	*Cambarellus sp. (*clade VI)	*Cambarellus*	JX127730	JX127604	JX127873	JX127465	JX127325
54	*Cambarellus sp. (*clade VI)	*Cambarellus*	JX127757	JX127631	JX127900	JX127492	JX127352
54	*Cambarellus sp. (*clade VI)	*Cambarellus*	JX127790	JX127664	JX127933	JX127525	JX127385
55	*Cambarellus montezumae*	*Cambarellus*	JX127731	JX127605	JX127874	JX127466	JX127326
55	*Cambarellus montezumae*	*Cambarellus*	JX127763	JX127637	JX127906	JX127498	JX127358
56	*Cambarellus montezumae*	*Cambarellus*	JX127776	JX127650	JX127919	JX127511	JX127371
56	*Cambarellus montezumae*	*Cambarellus*	JX127777	JX127651	JX127920	JX127512	JX127372
56	*Cambarellus montezumae*	*Cambarellus*	JX127778	JX127652	JX127921	JX127513	JX127373
57	*Cambarellus sp.* (clade VIII)	*Cambarellus*	JX127727	JX127601	JX127870	JX127462	JX127322
57	*Cambarellus sp.* (clade VIII)	*Cambarellus*	JX127767	JX127641	JX127910	JX127502	JX127362
58	*Cambarellus occidentalis*	*Cambarellus*	JX127784	JX127658	JX127927	JX127519	JX127379
58	*Cambarellus occidentalis*	*Cambarellus*	JX127785	JX127659	JX127928	JX127520	JX127380
59	*Cambarellus occidentalis*	*Cambarellus*	JX127813		JX127956	JX127548	JX127408
	*Procambarus toltecae*		JX127823	JX127687	JX127966	JX127558	JX127418
	*Procambarus acutus1*		JX127824	JX127688	JX127967	JX127559	JX127419
	*Procambarus acutus2*		JX127827		JX127970	JX127562	JX127422
	*Procambarus llamasi1*		JX127825	JX127689	JX127968	JX127560	JX127420
	*Procambarus llamasi2*		JX127826	JX127690	JX127969	JX127561	JX127421
	*Procambarus clarkii*		JX127829	JX127692	JX127971	JX127563	JX127424
	*Procambarus bouvieri*		JX127828	JX127691			JX127423
	*Orconectes deanae*		JX127859	JX127717	JX128000	JX127590	JX127451
	*Orconectes ronaldi*		JX127865	JX127722	JX128005	JX127596	JX127457
	*Orconectes virilis1*		JX127866	JX127723	JX128006	JX127597	JX127458
	*Orconectes virilis2*		JX127860			JX127591	JX127452
	*Cambarus brachydactylus* ++		DQ411732	DQ411729	DQ411783		DQ411802
	*Cambarus maculatus*		JX127864	JX127721	JX128004	JX127595	JX127456
	*Cambarus pyronotus*		JX127862	JX127719	JX128002	JX127593	JX127454
	*Cambarus striatus*		JX127861	JX127718	JX128001	JX127592	JX127453
	*Fallicambarus byersi*		JX127863	JX127720	JX128003	JX127594	JX127455
	*Fallicambarus caesius*		JX127867	JX127724	JX128007	JX127598	JX127459
	*Fallicambarus fodiens*		JX127858	JX127716	JX127999	JX127589	JX127450

+ Locality number, as depicted in Figure 1.

* Type specimens or type localities.

** Morphologically identified as *C. shufeldtii*.

*** Morphologically identified as *C. puer*.

++ Sequence from the study of Buhay et al. 2007, tissue originally from the Carnegie Museum of Natural History.

Populations termed as ‘*C. sp*’ are new proposed taxa, according to phylogenetic structure (see Figure 3).

Populations from clade I are included in the lineage of *C. zempoalensis*, species which has to be re-examined by incorporing *C. montezumae lermensis* in the analysis.

A series of apomorphic morphological characters define the subfamily and, therefore, monophyly has been accepted since its proposal. These include, as for other crayfish groups, genital morphology, which is particularly important, but also a small body size, specific branchial formula, movable and enlarged *annulus ventralis* (female genitalia) and the absence of the cephalic process in the first pair of pleopods (male genitalia) [1], [2], [6]. The morphological unity of these characters that define the subfamily contrasts with the wide morphological variation in other characters described for populations of several species [2], [5], [7]. This diversity within and among species makes designation and identification difficult, especially for widely distributed species [2], [5].

**Figure 2:**
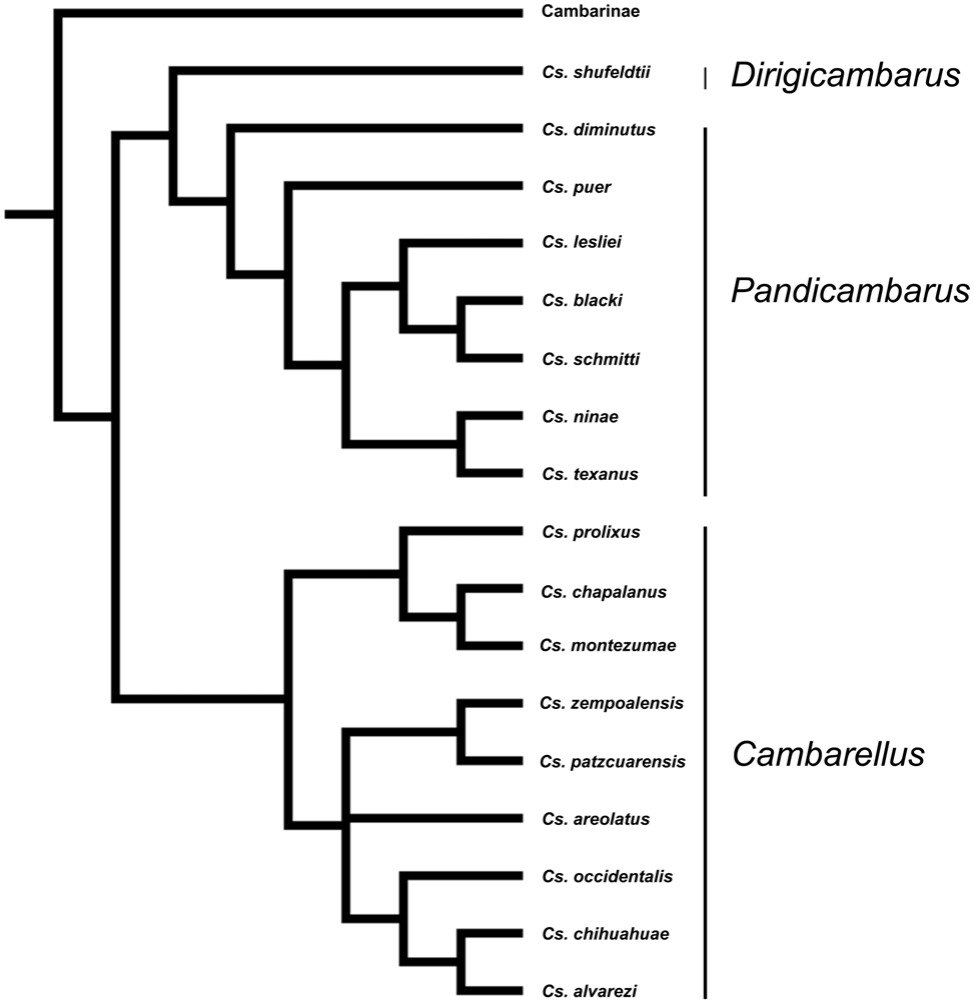
Morphologic hypothesis tested. Phylogenetic hypothesis based on morphologic analysis of the monotypic subfamily Cambarellinae (genus *Cambarellus*), indicated are the subgenera previously proposed, mainly based on genital morphology (Fitzpatrick, 1983).

Despite the intriguing geographic distribution and species diversity in the Cambarellinae, the only phylogenetic hypothesis for species relationships in the group is based on phenotypic information and genital morphology [2]. With this hypothesis (Fig. 2) three subgenera were proposed; *Pandicambarus* (containing seven species), the monotypic *Dirigicambarus,* both comprised of species occurring north of the Rio Grande (the Gulf Group), and *Cambarellus*, containing species south of the Rio Grande (the Mexican Group) [2]. However, no apomorphic characters have been proposed to support these subgeneric classifications and no formal phylogenetic hypothesis has been evaluated using either molecular or morphological characters. Therefore, we propose to estimate a robust phylogenetic hypothesis for the group using an extensive molecular data set. We then use this phylogenetic framework to evaluate a coherent taxonomy for the group and to test biogeographic hypotheses regarding the origin and spread of the dwarf crayfish.

We also examine diversification patterns in the subfamily through the estimated phylogenetic history of the species within the subfamily. Phylogenetic diversity patterns are impacted by geographic features and geologic history due to their effects on allopatric speciation [8]. Given the contrasting geographical features (Fig. 1) coupled with their distinct geological histories occupied by the different groups in Cambarellinae, we will use reconstructed molecular phylogenies to serve as models of lineages through time (LTT), that will allow us to test the tempo and pattern of change across lineages [9], [10], [11]. In the present study, we used our molecular dataset on the subfamily Cambarellinae to infer the timing and mode of lineage accumulation (patterns of speciation minus extinction) which allows us to determine whether there have been contrasting patterns in rates of diversification between the two geographical components of this group; namely, those defined as the Gulf and Mexican Groups, as a result of contrasting biogeographic histories. Finally, we identify a geological timescale consistent with biogeographic factors and cladogenetic events in this group.

## Materials and Methods

### Sampling and Sequencing

No specific permits were required for the described field studies, as none of the studied species were included in any endangered list, at national or international levels at the time of sampling (comprising the years 2005 and 2006). Including field and museum localities, 59 geographic locations covering 14 of the 17 species were collected throughout the distributional range of the subfamily Cambarellinae (Fig. 1). Taxonomic identification was carried out using existing keys [12]. The two main ranges for the subfamily were covered, along the Neartic and the Transition zone of North America, from the Mississippi River basin to the TMVB in central México. Most of the species could be sampled, but those tissues from species with very restricted distribution ranges and/or being collected in a reduced number of times in wild were obtained from museum specimens (National Museum of Natural History, Smithsonian Institution) (Table 1). Detailed data about samples included are summarized in the Table S1.

The central goal of this work is to estimate a robust phylogenetic hypothesis for relationships among the species within the subfamily to test taxonomic hypotheses, biogeographic hypotheses, and speciation hypotheses. As phylogenies are most accurately estimated using broad taxonomic sampling as well as extensive character sampling, we attempted to sample all species within the subfamily (but are missing three of them) and collected sequence data from five different gene regions (three mitochondrial and two nuclear). We sequenced the mitochondrial genes 16S rDNA (16S), 12S rDNA (12S) and Cytochrome Oxidase subunit I (COI). These genes have good phylogenetic signal in crustaceans [13] and are considered optimal choices to characterize the genetic variation in crustacean groups. Nuclear genes sequenced were 28S rDNA large ribosomal unit (28S) and Histone 3 (H3) gene, which also have some variation among species and are particularly good at discerning deeper nodes [13].

PCR amplifications using gene specific primers (Table 2) were carried out in 25 µL reactions containing: 1× PCR buffer, 0.5 µM of each primer, 0.2 mM of each dNTP, 1.5 mM MgCl_2_, 1 U *Taq* polymerase (Biotools), and about 10–50 ng of template DNA. The cycling profile for PCR amplifications was 3 min at 94°C (1 cycle), 30 s at 94°C, 30 s at the primer-specific melting temperature and 60 s at 72°C (30 cycles), followed by a final extension of 4 min at 72°C. PCR products were visualized in 1.0% agarose gels (1×TBE) and stained with SYBR-Safe (Invitrogen). Fragments were sequenced on an ABI 3730XL DNA Analyzer. Sequences of the different gene fragments were aligned using MUSCLE [14]. In the case of the COI gene, recommendations to detect the occurrence of possible nmtDNA were carried out for each sequence. These included the identification of stop codons, repeated sequencing of samples, nonsynonymous substitution and unusual levels of genetic divergence in samples from the same population [15], [16].

**Table 2:** Primer and PCR conditions used in this study to amplify different gene regions.

Generegion	primers	sequence	Tm(°C)	Reference
**COI**	COIAR	GTTGTTATAAAATTHACTGARCCT	48.5	This study
	COIBF	GCYTCTGCKATTGCYCATGCAGG	48.5	This study
	COIBR	TGCRTAAATTATACCYAAAGTACC	48.5	This study
	COICF	ACCTGCATTTGGRATAGTATCTC	48.5	This study
	COICR	GAAWYTTYAATCACTTCTGATTTA	48.5	This study
	COIDF	CTGGRATTGTTCATTGATTTCCT	48.5	This study
	ORCO1F	AACGCAACGATGATTTTTTTCTAC	48.5	[75]
	ORCO1R	GGAATYTCAGMGTAAGTRTG	48.5	[75]
**16S**	1471	CCTGTTTANCAAAAACAT	46	[76]
	16S-1472	AGATAGAAACCAACCTGG	46	[76]
**12S**	12sf	GAAACCAGGATTAGATACCC	53	[77]
	12sr	TTTCCCGCGAGCGACGGGCG	53	[77]
**28S**	28s-rD1a	CCCSCGTAATTTAAGCATATTA	52	[78], [79]
	28s-rD3b	CCYTGAACGGTTTCACGTACT	52	[78], [79]
	28s-rD3a	AGTACGTGAAACCGTTCAGG	52	[78], [79]
	28s-rD4b	CCTTGGTCCGTGTTTCAAGAC	52	[78], [79]
	28sA	GACCCGTCTTGAAGCACG	52	[78], [79]
	28S B	TCGGAAGGAACCAGCTAC	52	[78], [79]
**H3**	H3 AF	ATGGCTCGTACCAAGCAGACVGC	57	[80]
	H3 AR	ATATCCTTRGGCATRATRGTGAC	57	[80]

### Phylogenetic Analyses

Partition homogeneity tests were carried out on the concatenated matrix using PAUP v. 4.0b10 [17]. We examined homogeneity across partitions by gene and by codon position for protein-translated fragments (Table 3). We estimated phylogenies using Maximum Likelihood (ML) and Bayesian Inference (BI) approaches. Additionally, we used 15 species of the family Cambaridae as outgroups: *Cambarus maculatus*, *C. striatus*, *C. pyronotus*, *C. brachidactylus, Orconectes ronaldi*, *O. virilis*, *O. deanae*, *Fallicambarus caesius*, *F. fodiens*, *F. byersi*, *Procambarus bouvieri*, *P. clarkii, P. llamasi, P. acutus* and *P. toltecae* (Table 1).

**Table 3:** Substitution model and phylogenetic performance of each gene fragment.

Gene	Size (pb)	Substitution model/gamma parameter/Invariable sites		Variable sites	PI	%PI
		AICc	BIC			
**16S**	501	HKY+G; 0.232	HKY+G; 0.230	199	121	24.1
**12S**	358	K80+G; 0.219	TVM+G; 0.213	143	80	22.3
**COI**	1527	HKY+G; 0.321	HKY+G; 0.321	530	502	32.8
**28S**	992	TIM3+G; 0.031	TIM3+G; 0.031	39	28	2.8
**H3**	322	JC; –	HKY+I; 0.834	31	24	7.4
**All**	3700	GTR+G; 0.256	GTR+G; 0.254	1431	847	22.8

In order to identify the most appropriate evolutionary model of nucleotide substitution (Table 2), we considered the Akaike corrected information criterion (AICc) [18], and the Bayesian Information Criterion (BIC) [19] as estimated using the program jModeltest [20]. A phylogenetic tree was constructed under ML using PHYML 3.0 [21] and AICc-selected parameters for the concatenated matrix. The tree search was started with an initial BIONJ tree estimation followed by a Subtree Pruning and Regrafting (SPR) topological moves algorithm. We assessed confidence in branches using 1000 nonparametric bootstrap [22] replicates under the best-fit evolutionary model.

Bayesian inference of phylogeny was implemented in MrBayes v. 3.1.2 [23], following the BIC-selected parameters and applying a Monte Carlo Markov Chain (MCMC) search procedure for 10 million generations. Sequences were partitioned by codon position for COI and by gene for the rest of fragments, using the parameters found by BIC as priors and unlinking the run parameters. Convergence between the different run parameters in paired simultaneous runs (4 chains by run), trees were sampled every 100 generations and run length was adjusted considering an adequate sampling based on average standard deviation of split frequencies being <0.01 [24]. We examined the results and determined the burn-in period as the set of trees saved prior to log likelihood stabilization and convergence as estimated using Tracer 1.4.1 [25], eventually the first 10% trees. Tracer was also used to check for convergence between chain runs and optimal values of run parameters. Confidence in nodes was assessed from the posterior probabilities along the MCMC run. Highly supported nodes are termed herein as those with a value of 95% or more in posterior probabilities and bootstrap values.

We tested our resulting topology against the phylogenetic hypotheses put forth by Fitzpatrick [2]; namely, the three subgenera are monophyletic and show the following relationships ((*Dirigicambarus*, *Pandicambarus*),*Cambarellus*). Topology constrained ML scores were estimated for each hypothesis in PAUP*. Congruence with alternative hypotheses was evaluated in a ML framework applying the Shimodaira-Hasegawa (SH; [26]) test and the Approximate Unbiased (AU) test [27] with 50,000 RELL bootstrap replicates as implemented in TreeFinder [28]. We also tested these hypotheses using a Bayesian approach by identifying the alternative hypothesis within the set of Bayesian tree topologies and testing for significant differences. To do so, we filtered the post-burnin Bayesian topologies included in the set of trees with the constraint topology in PAUP* [17].

### Divergence Dating

In order to propose an accurate time frame for phylogenetic divergence processes, we estimated mean node ages and their 95% highest posterior densities (HPDs) using Bayesian relaxed molecular clock methods [29] as implemented in BEAST ver. 1.6.1 [30]. In this method, tests of evolutionary hypotheses are not conditioned on a single tree topology, which allows for simultaneous evaluation of topology and divergence times while incorporating uncertainty in both. A uniform Yule tree prior was specified, as appropriate for hierarchical rather than reticulate relationships, and a subsampling of one representative of every lineage was included to avoid over-representation of certain individual lineages with more sampling. We applied the optimal model of data partitioning and DNA substitution identified by BIC for each gene (COI, 16S, 12S, 28S and H3) and for codon positions for COI. An uncorrelated relaxed lognormal molecular clock was applied to model rate variation across branches, and pertinence of a relaxed estimation was checked after verifying that the distribution of the coefficient of variation was >1. The dating analysis was performed with the total matrix, but calibration of the molecular clock was done using COI and 16S mutation rates only, as information on rates of mutation of these two fragments is widely described in multiple groups and for which there is extensive fossil calibrated divergence time data in crustaceans [31], [32]. As a representation of these substitution rates, we considered the range to include extreme values reported, which extends between 0.23–1.1% per million years (PMY) for 16S [33], [34] and 0.7–1.3% PMY for COI [35], [36], [37]. These sets were introduced as uniform prior distributions, as no evidence justifies a specific distribution of rates in our data, avoiding the introduction any additional bias to the rate values assumed. Considering the geographic distribution of the genus, a geological calibration was also included as identified with the uplifting of the TMVB, which began around 12 MYA [38]. This age was set as a maximum for MRCA of the Mexican species. Additionally, fossil calibration was included in one point as the minimum age to account from the oldest fossil from the genus *Procambarus* [a *Procambarus primaevus*, 52.6–53.4 MYA, [39]]. Monophyly was not enforced for any node. Analyses were run for 20 million generations with a sampling frequency of 2000 generations. Tracer was used to determine the appropriate burn-in by monitoring run parameters by ensuring all effective sample sizes (ESS) were larger than 200 and independent runs converged. Two million generations were discarded before recording parameters and four independent runs were performed to ensure values were converging on similar estimates.

### Diversification Patterns

The two main components of the subfamily occupy two regions highly contrasting in topography and biogeographic history. Thus, a second objective in this study was to describe the patterns of cladogenesis involved in the evolutionary history of Cambarellinae and to test the hypothesis that the different biogeographic histories from the two different geographic ranges of the subfamily (i.e., the Mexican and Gulf Groups), could lead to contrasting cladogenetic patterns evidenced by possible diversification shifts. Shifts in birth and death rates can leave distinctive signatures in phylogenies, resulting in departures from linearity in semi-log LTT plots [9], [11]. We compared diversification rates from the reconstructed phylogeny of the entire subfamily and of the two main clades (Mexican Group vs. Gulf Group) to different null models of diversification by using the Birth-Death Likelihood method (BDL). This temporal method was used to test different hypothesis of cladogenesis rate shifts [40]. BDL uses maximum likelihood estimates of speciation rate parameters and a likelihood score per tree, and test different rate-variable models against null models of rate-constancy under the Akaike Information Criterion (AIC) [18]. To provide an indication of the diversification rates in each case, we generated a logarithm LTT plot using the LASER package version 2.2 [41]. The LTT plot was generated from the Maximum Clade Credibility tree from BEAST, after pruning the terminals not included in each clade tested using TreeEdit v1.0a10 [42] and rooting the basal age to the one observed from the dating analysis. To test for significant departures from the null hypothesis of rate-constancy, observed ΔAIC_RC_ from our data was compared to those from the different rate diversification models using BDL as implemented in the LASER package version 2.2 [41]. The test statistic for diversification rate-constancy is calculated as: ΔAIC_RC_ = ΔAICR_C_−ΔAICR_V_, where AICR_C_ is the Akaike Information Criterion score for the best fitting constant-rate diversification model, and AICRv is the AIC for the best fitting variable-rate diversification model. Thus, a positive value for ΔAIC_RC_ indicates that a rate-variable model best approximates the data. We tested five different models, of which two are rate-constant and three are rate-variable: 1) the constant-rate birth model (Yule) [the Yule process; [43]] with one parameter λ and μ set to zero; 2) the constant-rate birth-death model with two parameters λ and μ (BD); 3) a pure birth rate-variable model (yule2rate) where the speciation rate λ1 shifts to rate λ2 at time ts, with three parameters (λ1, λ2, ts); density-dependent speciation models with two variants, 4) exponential (DDX) and 5) logistic (DDL). Significance of the change in AIC scores was tested by generating a distribution of scores. This was done through simulation of 9000 trees using yuleSim in LASER, for the entire Cambarellinae subfamily and each geographic group, reflecting our sampling size in each case and having the same speciation rate as under the pure-Birth model.

## Results

### Phylogeny

We sequenced three mitochondrial (16S (519 bps), 12S (365 bps) and COI (1527 bps)) and two nuclear (28S 1100 bps and H3 322 bps) gene fragments resulting in 3833 characters (2411 mitochondrial and 1422 nuclear) and giving a series of substitution models (Table 3). These new data have been deposited in GenBank (Table 1). COI-like sequences were found in seven cases, identified by the occurrence of one or several stop-codons along the sequence and an unusual sequence divergence, which affected position in the tree and divergence regarding the other sequences coming from the same population. These sequences were removed from data sets and not considered for any analysis. As previously reported [15], when working with COI sequences in crayfish these sequences have to be specially checked to ensure they are mitochondrial.

The most variable fragment was 12S, followed by COI and 16S (variable sites: COI = 530/1527, 16S = 199/519 12S = 143/365; besides this, COI showed the highest proportion of parsimony informative (PI) sites: COI = 419, 16S = 121, 12S = 80) (Table 3). As expected, nuclear fragments were the most conservative (for the mitochondrial set, variable sites = 1187, PI = 783; for the nuclear set, variable sites = 244, PI = 64). The complete combined data set contained 1431 variable sites (∼37%), and 847 PI (∼22%).

**Figure 3:**
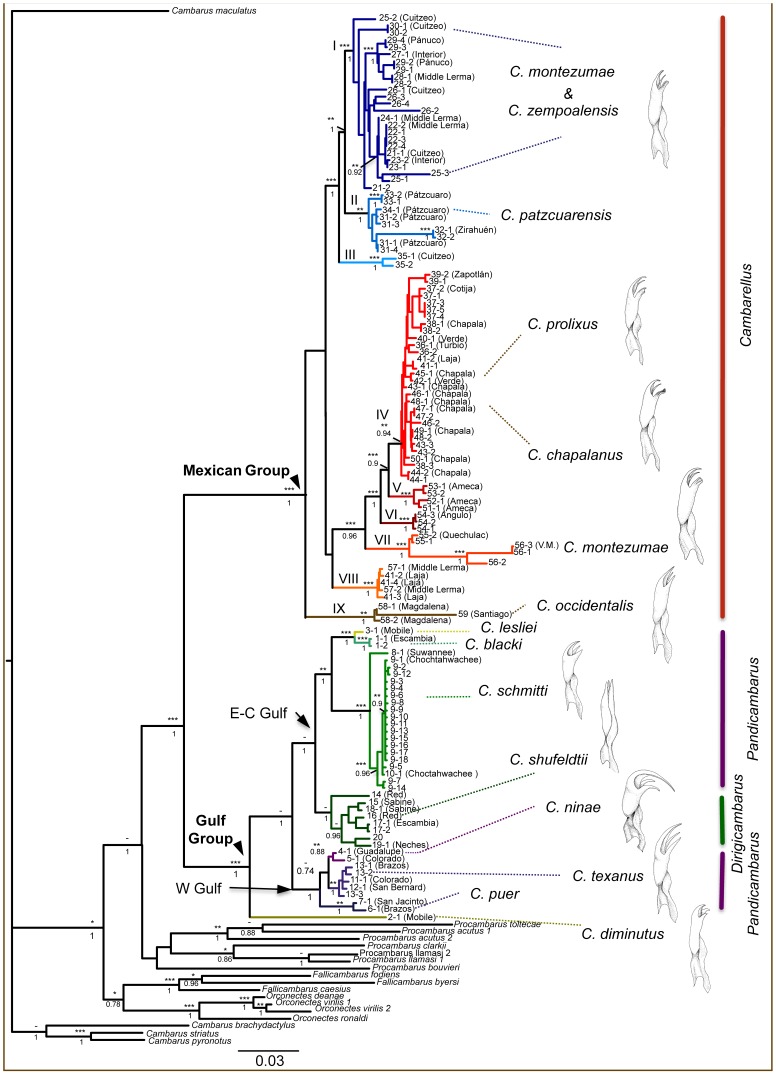
Phylogenetic tree of *Cambarellus* genus. Phylogenetic tree of *Cambarellus* based on three mitochondrial and two nuclear genes. Bootstrap support from ML (above) and Posterior Probabilities from Bayesian Inference (bellow) are indicated on each node. ***Stands for 95 or more, **for 85–94 and *for 75–84 support values from ML analyses. Drawings correspond to male genital morphology, which is the base for traditional taxonomy of subgenus and species in the group. Individual 5–1 was morphologically identified as *C. shufeldtii*, but is considered here as *C. ninae* based on the phylogenetic position in tree.

The topologies recovered by mitochondrial and nuclear analyses based on ML and BI methods were similar (Figure 3), although some discrepancies can be found in some terminal taxa arrangements and between genera-outgroup relationships, principally concerning the relative positions of Cambaridae genera representatives. Both topologies show *Cambarellus* as a monophyletic clade (Figure 3). Within *Cambarellus* we found two divergent clades which correspond to the two distinct geographic ranges of the genus based on a highly supported node by ML and BI analyses (more than 95% of nodal support values). The first lineage included the species from the Mexican Group, coincident with the TMVB in México. The second lineage included the Gulf Group, containing the species distributed in USA. Only results from the combined analyses of mitochondrial and nuclear information are shown, as nuclear evidence did not have enough phylogenetic signal to distinguish relationships within each geographic group (Mexican and Gulf Groups). As shown in different studies, mitochondrial and nuclear information could resolve different portions of the phylogeny (i.e., shallow vs. deep levels of tree, [44], [45] ) and that was one of the major reasons for combining these data types in this study. The hypothesis explaining this is that long-branch attraction might be more common among deeper nodes, and that slow-evolving nuclear DNA might help to resolve such issues [46], [47].

Topology tests rejected the null hypothesis of an equally good explanation for all the constrained and the unconstrained topologies. The topology obtained in this study showed a significantly better Likelihood score (L = −27483.1) than the monophyletic grouping of *Pandicambarus* subgenus. Our phylogenetic estimate resulted in a monophyletic subgenus *Cambarellus* and *Dirigicambarus*, but *Dirigicambarus* was nested within the paraphyletic *Pandicambarus* (Fig. 3). We tested the monophyly of the *Pandicambarus* by forcing this alternative topology and we can reject this hypothesis by the results of SH and AU tests (likelihood values for the alternative hypothesis/p values for SH and AU - 27565.1/0.043, 0.047). Except for the division within *Pandicambarus*, Fitzpatrick’s notion of relationships among the subgenera is supported by our resulting topology, except for the non-monophyletic *Pandicambarus* as *Pandicambarus* and *Dirigicambarus* are nested together as a sister clade that is then sister to *Cambarellus* as proposed by Fitzpatrick. Bayesian inference also failed to support the monophyly of *Pandicambarus* failing to find a monophyletic *Pandicambarus* in 9900 trees resulting from the MCMC search.

Species were generally well recovered as monophyletic groups for most of those included in the Gulf Group, but a different situation is depicted for the Mexican Group (Figure 3). The clades highly supported by phylogenetic analyses have a geographic concordance, supporting the hypothesis that geographic events could have been important factors influencing cladogenesis in the genus, especially those regarding geographic features of the TMVB. Phylogenetic structuring between all Mexican taxa did not support the monophyly of some of the species currently recognized, as the highly supported clades showed representatives of multiple named species, suggesting that some of the named species did not form monophyletic assemblages.

Low 16S divergences can be observed between taxa. Divergences obtained between those contained in the Gulf Group were higher than those from the Mexican Group. The mean sequence divergence considering the likelihood model within the former was D_HKY_ = 4.13%, and that within the latter was D_HKY_ = 1.18% (Table 4).

**Table 4:** Uncorrected (below diagonal) and ML 16S rDNA distances (above diagonal) between phylogenetic groups of *Cambarellus*.

	1	2	3	4	5	6	7	8	9	10	11	12	13	14	15	16	17	18	19	20	Within Group unco	Within Group HKY
***C. zempoalensis*** **Lerma-Cuitzeo**		0.33	0.98	1.17	0.89	2.33	1.57	2.58	1.25	12.11	11.25	10.9	10.04	11.63	11.38	13.01	15.91	18.47	18.98	23.74	0.16	0.16
***C. patzcuarensis***	0.32		1.13	1.41	1.13	2.7	1.93	2.96	1.58	11.72	10.88	11.15	10.51	11.82	11.55	12.76	15.56	17.94	18.41	22.73	0.13	0.13
***C. sp.*** ** LaMintzita**	0.93	1.07		1.53	1.36	2.9	1.97	3.13	1.75	12.67	11.8	11.24	10.63	12.15	11.9	14.31	17.12	19.31	18.71	24.64	1.24	1.3
***C. chapalanus*** ** Chapala**	1.09	1.29	1.42		0.77	1.91	1.31	2.04	1.01	12.17	11.31	10.79	9.43	10.89	10.62	12.79	15.56	18.84	17.12	26.92	0.64	0.68
***C. sp.*** ** Ameca**	0.85	1.07	1.28	0.74		2.01	1.2	2.24	0.83	13.8	12.81	12.23	10.64	12.24	11.66	14.64	17.41	20.2	19.11	30.08	0.11	0.11
***C. sp.*** ** Zacapu**	2.08	2.37	2.57	1.74	1.85		1.87	1.14	1.81	12.44	11.55	11.7	10.94	12.29	11.37	14.13	17.29	20.6	18.79	29.79	0.58	0.6
***C. montezumae*** ** Xochimilco**	1.43	1.72	1.8	1.21	1.13	1.7		2.15	0.95	12.24	11.35	11.54	9.5	10.91	10.56	12.79	15.33	18.9	18.07	29.03	0.31	0.32
***C. sp.*** ** Vegil**	2.31	2.6	2.77	1.86	2.06	1.09	1.97		1.82	12.25	11.36	11.02	10.22	11.41	10.41	13.59	15.98	18.79	17.73	29.43	0.00	0.00
***C. occidentalis***	1.16	1.45	1.61	0.95	0.8	1.67	0.9	1.69		12.56	11.67	12.07	9.57	10.95	10.55	13.24	15.1	19.11	17.88	29.03	0.14	0.14
***C. lesliei***	8.27	8.11	8.57	8.3	9.09	8.45	8.35	8.3	8.48		0.41	3.26	2.76	3.09	3.16	4.89	9.64	12.25	13.1	20.47	0.00	0.00
***C. ninae***	7.85	7.68	8.15	7.87	8.63	8.01	7.91	7.86	8.04	0.41		2.75	2.26	2.65	2.64	4.32	8.89	11.53	12.51	19.73	0.00	0.00
***C. schmitti***	7.73	7.85	7.94	7.68	8.44	8.1	8.05	7.66	8.27	2.85	2.44		3.89	4.62	4.66	6.34	10.94	14.47	15.49	20.77	0.00	0.00
***C. shufeldtii*** ** La Fayette**	7.21	7.43	7.54	6.9	7.58	7.72	6.96	7.25	6.97	2.44	2.04	3.26		1.82	1.82	4.45	7.99	13.31	14.55	20.55	0.00	0.00
***C. shufeldtii***	8.03	8.12	8.31	7.69	8.39	8.42	7.72	7.9	7.73	2.74	2.39	3.84	1.68		1.51	4.54	8.41	13.06	13.54	22.04	1.58	1.69
***C. texanus***	7.91	7.99	8.19	7.55	8.11	7.99	7.53	7.41	7.5	2.79	2.36	3.85	1.67	1.42		4.8	7.42	12.11	13.25	22.08	0.85	0.88
***C. puer***	8.51	8.41	9.09	8.42	9.27	9.06	8.45	8.71	8.62	4.01	3.6	4.89	3.62	3.76	3.92		7.02	13.75	15.46	20.96	0.80	0.72
***C. diminutus***	9.96	9.84	10.43	9.82	10.6	10.56	9.75	9.92	9.62	6.92	6.5	7.52	5.91	6.25	5.64	5.37		16.3	15.91	25.11	0.00	0.00
***Procambarus***	10.78	10.61	11.15	10.92	11.42	11.57	10.95	10.89	10.99	8.48	8.11	9.42	8.87	8.86	8.39	9.05	10.04		14.51	22.17	7.31	10.84
***Orconectes***	11.1	10.92	11.09	10.5	11.27	11.16	10.8	10.81	10.76	8.89	8.67	9.95	9.55	9.16	9.06	9.92	10.17	9.36		20.52	0.00	0.00
***Cambarus***	12.78	12.51	13.06	13.6	14.55	14.38	14.13	14.26	14.14	11.65	11.43	11.82	11.69	12.29	12.22	11.85	13.14	11.95	11.67		0.00	0.00

The Mexican Group is composed of several clades highly supported by ML and BI analyses (95–100% support, termed with roman numerals in Figure 1), which also show geographic concordance. Some geographic overlapping between clades was observed, mainly along the Lerma Basin. The Clade I included populations from the Cuitzeo and Middle-Lerma basins, morphologically assigned to *C. montezumae*. *C. zempoalensis* from type locale was placed inside this clade as well. *Cambarellus patzcuarensis* from the basins of Pátzcuaro and Zirahuén were contained in Clade II and sister clade to Clade I. The third and more divergent clade (Clade III) consisted of a population from La Mintzita, geographically close to the Cuitzeo basin.

Clade IV consisted of populations from the basin of Chapala and its tributaries (Duero River), as well as its neighboring basins, Cotija and Zapotlán. This group included two species, *C. chapalanus* and *C. prolixus*, both found in Lake Chapala and associated with different habitat conditions. Also included here were populations from up-stream tributaries of the Santiago River, which originates as an outflow of the Chapala Lake. Clade V contained populations from the river Ameca basin. Clade VI contained the population from Zacapu Lagoon. The Clade VII included two populations from the eastern-limits of the distribution of the genus in the TMVB, the populations of Xochimilco (type locality for *C. montezumae*) from the Valley of México basin and the crater lake Quechulac. The Clade VIII was composed of two populations from the northern margin of the Middle-Lerma basin and the Clade IX by populations from the basins of the Santiago and Magdalena rivers, in the west part of TMVB.

Gulf Group relationships depict a phylogenetic structuring corresponding to geographic ranges. *C. diminutus* corresponds to the most divergent lineage, while two clades were recovered with high ML and BI support corresponding to a west-east pattern. The first clade contained most of the species from the Central and East Gulf Coast (CEG), except *C. diminutus*, and included four recognized species. Populations of *C. shufeldtii* from the Mississippi river basin form a monophyletic group, while *C. blacki*, *C. lesliei,* and *C. schmitti* are grouped together in a sister clade to the latter, geographically covering the eastern extreme distribution range of the genus in the Gulf Group from the Mobile Bay, Alabama to the Swuanee River, Florida. A similar grouping is observed in the second clade of the Gulf Group, containing populations from the West Gulf Coast (WG), mainly in the south-west part of Texas, where *C. puer* was recovered as a sister lineage to the clade grouping *C. texanus* and *C. ninae*.

### Diversification Patterns and Dating

**Figure 4:**
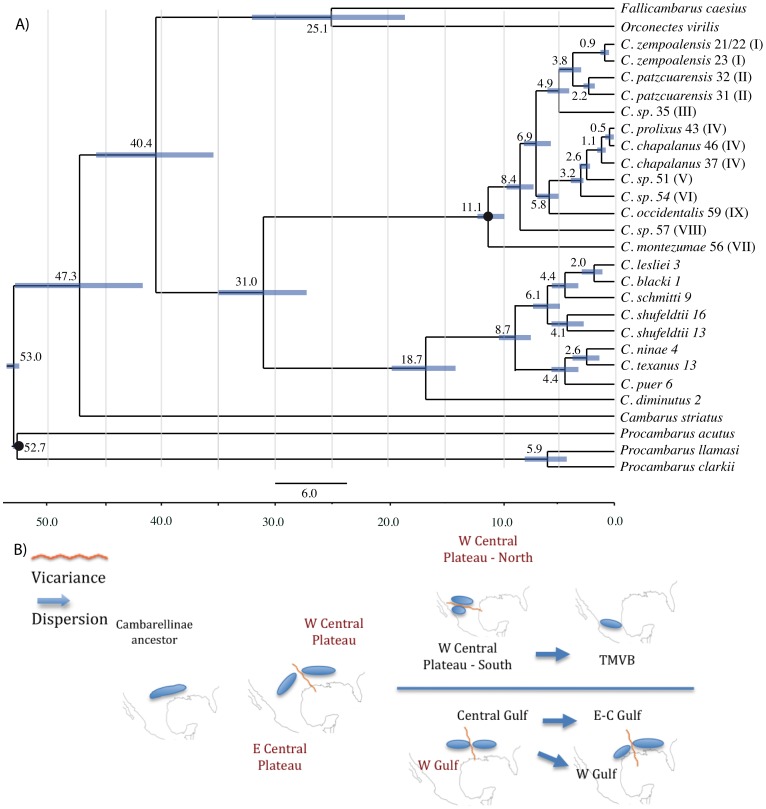
Molecular dating of cladogenetic events. Dates and major biogeographic events inferred during cladogenesis of the Cambarelline subfamily. A) Ultrametric tree resulting from the dating analysis. Mean ages are indicated in each node (MYA), and 95% HDP intervals are shown as blue bars. Black dots indicate node used for calibration (oldest fossil recorded for *Procambarus*). Numbers correspond to localities and roman numerals to clades from phylogenetic tree (Figure 3). B) Major cladogenetic events inferred from phylogenetic structure and dating. Red names refer to extinct lineages.

Log-likelihood scores with the molecular clock enforced and not enforced were −13.893 and −13.767, respectively. As the LRT rejected the null hypothesis of a global molecular clock (χ2 252, P = 0.001), the sequences analyzed did not evolve at a homogenous rate along all branches and we proceeded to use a relaxed molecular clock (Fig. 4) as a result.

Ages from the dating analysis were recovered with consistency through repetitions (Figure 4). The crown age for the tree was 53 Myr (95% highest posterior density [HPD] interval for node heights/ages: 52.6–53.7 Myr), which corresponds to the separation of the genus *Procambarus* from the rest of the groups. We estimated an approximate age of 31.0 Myr (27.4–34.9 Myr 95% HPD) for the TMRCA of clade containing the Cambarellinae. MRCA for the terminals included in the two lineages of the Gulf Group is approximately 16.7 Myr (13.9–19.7 Myr 95% HPD). MRCA of the Mexican Group was dated around 11.1 (9.8–11.9 Myr 95% HPD). We propose some major biogeographic events inferred from the phylogenetic structure, which depicted different vicariant and dispersion events along the evolutionary history of Cambarellinae (depicted in Figure 4.).

**Figure 5:**
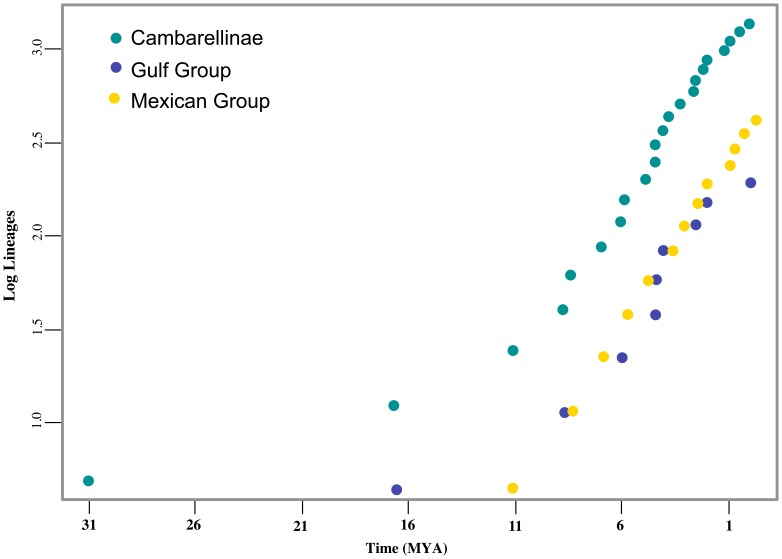
Diversification patterns through time. LTT plot for the Cambarellinae subfamily (green), the Mexican Group (yellow) and the Gulf Group (blue).

The LTT plots track the temporal accumulation of lineages in a clade and indicate that the subfamily Cambarellinae did not significantly deviate from a constant model of diversification during its evolutionary history, as evidenced in the LTT analyses for the entire subfamily (including both, Gulf and Mexican Groups, see Fig. 5). LTTs rate-constancy models received better AIC scores, and they were not significantly different from the best rate-variable model for all analyses (Table 5). The pure birth speciation rate model was identified as having the lowest AIC value amongst the other models tested for the subfamily together and the two groups separately. Although the Mexican Group showed the highest diversification rate (under pureBirth model r = 0.174), it is still a low value as compared to recognized shifts in diversification in other animal groups ranging from 0.4 to 0.8 speciation events per million years [48], [49].

**Table 5:** Results of the Birth-Death Likelihood analysis based on fitting different diversification models to Cambarellinae and its containing groups (Gulf and Mexican Groups).

Group		pureBirth	BD	DDL	DDX	yule2rate
**Cambarellinae**	Parameters	r1 = 0.122	r1 = 0.053	r1 = 0.122	r1 = 0.064	r1 = 0.043
			a = 0.706	k = 476707.6	x = −0.297	r2 = 0.152
	Ln(L)	16.600	15.815	16.601	15.969	14.668
	AIC	−35.201	−35.631	−37.201	−35.939	−35.336
	ΔAIC	0	−0.430	−2	−0.738	−0.135
**Gulf**		r1 = 0.106	r1 = 0.088	r1 = 0.197	r1 = 0.127	r1 = 0.153
			a = 0.229	k = 12.126	x = −0.117	r2 = 0.059
						st = 4.116
	Ln(L)	12.101	12.089	11.783	12.083	11.395
	AIC	−26.202	−28.179	−27.567	−28.166	−28.791
	ΔAIC	0	−1.977	−1.365	−1.964	−2.589
**Mexican**		r1 = 0.174	r1 = 0.174	r1 = 0.276	r1 = 0.296	r1 = 0.210
			a = 0.0	k = 21.007	x = 0.283	r2 = 0.120
						st = 2.225
	Ln(L)	10.201	10.201	9.894	10.049	9.834
	AIC	−22.402	−24.402	−23.789	−24.096	−25.669
	ΔAIC	0	−2	−1.387	−1.694	−3.267

r = net diversification rate (speciation events per million years);

a = extinction fraction;

st = time of rate shift (MYA);

k = carrying capacity prameter;

x = rate change parameter;

Ln(L) = Log-Likelihood;

AIC = Akaike information criterion;

ΔAIC = change in AIC relative to pureBirth.

Quick inspection of the LTT plots shows some differences between the cladogenesis of the entire subfamily and that of the Gulf and Mexican Groups alone (Figure 5). However, according to the BDL analysis, the diversification rate-constancy statistic ΔAICRc was found to be similar between them, being −0.135 for the entire subfamily, −1.38 for the Mexican Group and −1.36 for the Gulf Group, indicating that the data are a better fit to the constant rather than variable rate model of diversification in all cases. Goodness-of-fit tests indicated that the mean Bayes LTT from the entire subfamily was not significantly different from expectations under any of the rate constancy models (AIC pureBirth and BD = 35.20 and 35.63, respectively). The values from the BDL analysis of the Mexican and the Gulf Groups were not significantly different than the critical values found under the different simulated constant rate models (for AIC pureBirth = 22.40 and BD = 24.40 for the Mexican Group and AIC pureBirth = 26.20 and BD = 28.17 for the Gulf Group). These results are consistent with a lack of evidence about episodes of shifts in diversification rates along the evolutionary history of Cambarellinae or its two groups separately.

## Discussion

### Phylogenetic Relationships

Our results are consistent with the monophyly of the Cambarellinae subfamily, previously proposed from morphology and a set of apomorphic characters [2]. The combination of mitochondrial and nuclear markers provide sufficient information to resolve the relationships between highly supported clades, namely the Gulf (*Pandicambarus/Dirigicambarus*) and Mexican (*Cambarellus*) Groups and included clades (Figure 3). Less resolution is observed at the deeper nodes of the Mexican Group, where several clades were not supported by all analyses. It is possible, as commonly argued for polytomies, that such patterns could be related to an acceleration of speciation rates in a short period of time [50]. Species sampling in this study is not complete, as three species are still to be added to the phylogenetic analysis. These correspond to *C. alvarezi*, *C. areolatus* and *C. chihuahuae* from North of Mexico and have almost no collection records. Populations from the aforementioned species are currently under serious threat or possibly extinct, as we did not find any specimens in our attempts to collect them. Their rarity is possibly due to extreme habitat alteration or drought, a situation reported as critical for freshwater fauna in some of the localities from where they have been recorded [51], [52]. Their future inclusion, if possible (mostly through museum collections or captive populations), could provide valuable insight into the phylogenetic relationships within the subfamily, especially between the Mexican and Gulf Groups defined here.

Several differences can be found between the phylogenetic relationships emerging from this work and the previous hypothesis [2]. First, relationships between species in the Gulf Group are not congruent with several assumptions made from morphology, especially regarding the phylogenetic meaning of genitalia variation. Although species are generally well recovered as monophyletic, their relationships are not congruent. As evidenced by topology tests carried out in this study, sister relationships proposed by genital morphology between the two subgenera from the Gulf Group (*Pandicambarus* and *Dirigicambarus*) is not supported. Instead, *Dirigicambarus* (composed by *C. shufeldtii*) is recovered as a sister taxon of a clade containing *C. lesliei* and *C. schmitti*. This would leave the subgenus *Pandicambarus* as paraphyletic, ultimately questioning also its phylogenetic validity. Maintaining of the subgenus *Dirigicambarus* for *C. shufeldtii* could be also questioned, as no phylogenetic evidence supports it, pointing out that genital distinctiveness in this species could be the result of drift events or selective processes along its history. Besides its proposition as a member of a separate subgenus, *C. shufeldtii* has been recognized as a derived rather than a plesiomorphic representative [2], an assumption supported in this study. Therefore, we recommend that the subgenus *Dirigicambarus* be disregarded and that the genus *Cambarellus* should contain only two subgenera, namely *Cambarellus* and *Pandicambarus* that correspond to the Mexican and Gulf clades, respectively (resulting in *Cambarellus shufeldtii* being considered a member of the subgenus *Pandicambarus*). Our phylogenetic results support the hypothesis of *C. diminutus* as having plesiomorphic character states for the Gulf Group. Its unique morphological traits (outlined in [2]) are in agreement with this hypothesis.

### Taxonomic Implications

Numerous species concepts have been proposed that emphasize different features for delimiting species. Sometimes, this has led to contrasting conclusions regarding species limits and the number of species in many groups. A ‘unified species concept’ was advocated that emphasizes the common element found in many species concepts, which is that species are separately evolving lineages [53]. This unified concept also allows the use of diverse lines of evidence to test species boundaries [e.g., monophyly at one or multiple DNA loci, morphological diagnosability, ecological distinctiveness, etc. [53], [54] and is the species concept we follow in this study.

There were two cases in which the inferred topology did not recover species’ monophyly in the Gulf Group. The first one shown by one individual morphologically assigned to *C. shufeldtii* (Locality 5, Colorado Basin), which grouped with individuals of *C. ninae* and the other by one individual morphologically assigned to *C. puer* (Locality 12, San Bernard Basin), grouped with individuals of *C. texanus*. The most plausible explanation for this could be the finding of introgression of *C. shufeltii*, supported by the overlapping ranges of these species in east Texas. As a common consequence, introgression between species with smaller ranges could be favored when they share similar regions with widely distributed species like *C. shufeldtii* and *C. puer* (e.g., [55]). The aforementioned hypothesis needs to be supported with faster-evolving nuclear markers, which allow the differentiation between species, and could be approached in the near future.

For the Mexican Group, the phylogenetic structure shows a geographic correspondence. This observation supports the hypothesis that cladogenesis in the group has been influenced by geological history. This geographic correspondence could explain why instead of recovering species, cladogenetic structure recovered different hydrological units as monophyletic. This is the case for the widely distributed *C. montezumae*, which is not recovered as monophyletic, as several populations morphologically assigned to this species were located in different clades in the Mexican Group. In fact, several populations morphologically assigned to *C. montezumae* form a paraphyletic group, as *C. zempoalensis* is recovered inside this group. Another example concerns *C. prolixus*, included inside the wider genetic variation of *C. chapalanus*. However, the striking morphological distinctiveness of *C. prolixus* suggests a very recent processes of divergence between this species and *C. chapalanus* which may be missed by the genetic markers used here [see [56] for discussion on the relative importance of genetic markers versus selected morphological differences in species studies]. Based on an unified species criterion, we found support for all described species in the Mexican Group from TMVB, which match to the terminal clades in tree (Figure 3) plus *C. prolixus*, which possesses contrasting morphological and ecological features. These clades correspond to six described species: 1) *C. zempoalensis*, corresponding to the population from Zempoala. Temporarily, we consider this species as valid but this needs to be confirmed with an analysis including the ‘lermensis form’ (in the terms of Villalobos’ proposal) [57]. This is because when considering the range of this clade, it probably includes the aforementioned form, from the upper Lerma Basin. As such, *C. montezumae lermensis* would be raised to species rank and *C. zempoalensis* would stand as a junior synonym; all populations found in clade I would temporarily correspond to *C. zempoalensis,* until confirmation of the above mentioned issue regarding its synonymy with *C. montezumae lermensis*; 2) *C. patzcuarensis*, for those populations from the Patzcuaro basin; 3) *C. chapalanus*, from the basin of Chapala and adjacent basins; 4) *C. prolixus*, from certain habitat conditions at Chapala Lake; 5) *C. montezumae*, from the Valley of Mexico and adjacent basins and 6) *C. occidentalis*, from the lower part of the Río de Santiago basin, at the western extreme of the distribution in México. In addition, we found several monophyletic clades, and in congruence to the same criterion, we propose they correspond to no recognized species, those from the terminal clades in tree (Figure 3): 1) clade III, for the population from La Mintzita spring; 2) clade V, for populations from Ameca basin; 3) clade VI, for populations from the Zacapu Lagoon and 4) clade VIII, for certain populations from the northern side of the Middle Lerma Basin (populations of La Laja basin and Vegil, see Table S1).

### Rates of Cladogenesis and Contrasting Cladogenetic Forces

Unlike the cladogenetic structure, the rate at which cladogenesis took place in *Cambarellus* does not seem to be affected by geologic events. Even when most of the cladogenetic events in the Mexican Group are probably the result of vicariance corresponding to geological features as the formation of the TMVB, a geologic region that has been proposed to affect cladogenesis in different freshwater groups [58], [59], this study has found no effect of geologic events on speeding or reducing cladogenesis rates. Although different in nature, and affected by contrasting geographic ranges, vicariant events in both groups lead to similar cladogenetic trajectories, demonstrating the impact of climatic and geologic forces on allopatric speciation.

All these lines of geological evidence indicate that the historical geographic range of the hypothesized ancestral species of Cambarellinae in México and the Southeast of the United States have changed dramatically over time. Additionally, some other effects could have played a roll in speciation in both groups. Although the continental ice sheets during the Pleistocene glacial periods in North America never extended into the study area, these glaciations had some profound indirect effects in freshwater faunas in México and are hypothesized to have permitted dispersal by stream captures, local inland or estuarine flooding, and interconnecting drainages due to lowered sea levels during the late Neogene [60].

### Biogeography

Our results support that MRCA for the Cambarellinae existed in the Eocene, ∼40.4 MYA (35.2–45.7 MYA). A singular biogeographic event inferred from this study comes from the separation of the two major clades, which could be related to the Eocene-Oligocene boundary, a transition documented to strongly affect terrestrial, marine and freshwater dwellers, as evidenced by significant extinctions and taxonomic turnovers in a wide range of groups [61], [62]. In this case, the formation of the Rio Grande Rift could have vicariant effects on the ancestors of both groups.

We postulate that historical vicariant events are related to change in geographical barriers and climate in both groups while dispersal events of some species are responsible for occupying the current wider range, with their current absence related to extinction periods. While these biogeographic events are present in both groups, contrasting vicariance and dispersal impacts on distributions are not unusual for freshwater crayfishes [63]. Here we explain some possible alternatives, inferred from congruence in timing of cladogenetic events. Species cladogenesis in the Gulf and Mexican Groups are best explained by an allopatric mechanism of speciation because no overlap is observed between sister taxa in *Cambarellus* [8]. Estimation of divergence times provides a temporal scenario of these events, allowing for a relationship of earth history with hypothesized vicariant mechanisms proposed to promote allopatric speciation. We postulate that divergence patterns in these groups are contrasting in several ways. First, date estimates agree with a more ancient diversification in the Gulf Group than in the Mexican Group, even when possible events related to species diversification from Northern Mexico could not be inferred. It is possible that the latter predate the ones observed for the Mexican Group. Second, while the Gulf Group cladogenesis could be more related to climatic oscillations, orogenic impacts could have been more important for diversification of the extant species in the Mexican Group. The absence of *Cambarellus* from the Río Grande basin could be explained by a generalized extinction of its populations related to the high desiccation rate since the Tertiary [64]. The ultimate evidence of that would be the presence of *C. chihuahuae* from the Guzman basin in the Southern part of the Río Grande Rift. This high extinction rate could explain the current disjunct geographic pattern between the Mexican and Gulf Groups. It seems reasonable to consider that as a consequence of the extinction rate along the former contact zone between the groups. It would not be surprising to find relict populations from both groups if further sampling efforts in this region could take place, which could modify their known range and find regions containing both lineages.

### Proposed Vicariant Events Promoting Speciation

The first diversification event in the Gulf Group was dated to Early Miocene, ∼16.7 MYA (13.9–19.7 MYA), and corresponded to the separation of the *C. diminutus* lineage. It is possible that extinction events could explain the observation of a unique well differentiated branch leading to *C. diminutus*, although a wider genetic variation not yet sampled from this lineage could be possible, which would be consistent with the wide morphological variation previously observed [2]. Orogenic activity dating to this period corresponds at the SE of United States with the formation of the Edwards Plateau, and the Miocene increased activity along the Balcones Fault. The next cladogenesis recorded for the Gulf Group is congruent with Late Miocene times, approximately 8.7 MYA (7.3–10.3 MYA), originating the Western (WG) and Eastern Gulf Coast (EG) species groups. These speciation events are consistent with sea levels along the gulf coast driven by climatic oscillations since the Middle Miocene. These were characterized by a dramatic rise in sea level between 80 and 100 m above the present day sea level [65], [66]. As a consequence, a marine incursion took place along the coast of the Gulf of Mexico, which could be important in the split of West and Central-East distribution ranges and keep them separated long enough to induce a strong speciation event.

In the Late Miocene there was a sharp drop of 80–100 m below present sea levels, extending Gulf of Mexico tributaries further south. This southward extension of Gulf Coastal rivers created connections between tributaries that were isolated during periods of higher sea level. The Late Miocene drop in sea level correlates with the estimated age of the first speciation events among the extant species of *Cambarellus* from the Gulf Group. Later, the Pliocene (2.5–5.5 MYA) was characterized by a 50–80 m rise above current sea level, but this incursion lasted for only a short time, approximately one million years [66]. Sea levels dropped in the Late Pliocene, and during the Pleistocene there were at least three major fluctuations in sea level, none rising higher than 10–20 m above the current level [66], [67]. All these events could affect the most recent speciation events and possible inter-basin connection between the Gulf species of *Cambarellus* could allow for dispersal of some of the today widely distributed taxa along the coastal drainages.

As a lentic-habitat dweller is the widespread way of life for the subfamily, it is reasonable to think that this could be the same situation for the ancestors of the different groups. In this case, formation of Paleolakes during the Middle Miocene (∼10.8 MYA) along the Northern Central Plateau of México and South-East United States could be important features driving early cladogenesis in the Mexican Group.

The pattern of distribution observed in *Cambarellus* agrees with those proposed for other freshwater organisms, like the Plateau Track and western Mountain Track [64]. To explain similar distributions in fish genera such as *Ictalurus*, *Moxostoma,* and *Micropterus*, these patterns suggest former hydrographic exchanges across the present arid plateau. Based on faunal composition and the finding of sister taxa between those regions like Tampichthys/Codoma and Algansea/Agosia sister pairs [68], possible connections between drainages of the South Western Gulf Slope (Nueces, Colorado and Guadalupe rivers) and those from the northern Río Grande tributaries have been suggested [69]. These connections could explain the presence of the Northern Central Plateau species (*C. alvarezi*, *C. areolatus* and *C. chihuahuae*), especially joined to lake habitats. Extensive lakes associated with the past Río Grande inflow have been documented to cover much of north-western Chihuahua and southern New Mexico in Pleistocene times, like the Lake Cabeza de Vaca [70].

The reduction in volume of lacustrine habitats in the Central Plateau by climatic events may have resulted in a high rate of late Cenozoic extinction [64], and the patchy distribution pattern of *Cambarellus* in this region. This high rate of desiccation, now increased by human activities [51], could have eroded diversity in this region, driving to extinction most of the *Cambarellus* populations in the Northern Central Plateau of México and could also explain the current absence of *Cambarellus* from the rivers south of the West Gulf Coast drainages. Partial extirpation from a formerly continuous range due to increasing dry rate during the Tertiary, has also been seen in different fish groups with a similar range, like Goodeidae and Cyprinodontidae [71]. Additionally and continuing southward, former connections between Northern and Western Central Plateau rivers could explain the presence of *C. occidentalis* in the Lower Santiago basin and from there a connection to the rest of the TMVB could be inferred.

Along the TMVB, the Lerma-Santiago river system is the main drainage. Previous connections between the Lerma River and northeastern and western drainages have been suggested for Goodeidae and Cyprinid fish [71]. Similar to what has been postulated for freshwater fish groups like the families Atherinidae, Goodeidae Cyprinidea, diversification in *Cambarellus* along the TMVB could have been related to an ancient and successive fragmentation of the Lerma-Santiago drainage across extensive lacustrine systems from the Miocene to Pleistocene [71], [72].

Separation of the main clades of *Cambarellus* in the TMVB is dated along the late Miocene and Pliocene (10.8–4.6 MYA), a period of high geological activity in México [73]. Formation of the TMVB advanced in a West-East direction [74], and this could influence the separation of clades from the main groups of *Cambarellus*. This formation could have begun before the presence of *Cambarellus* in TMVB, given its absence on the Pacific basins south to the Zapotlán basin, at its western margin. Major diversification of the genus took place in an interval of time of less than 9 MYA.

This study found evidence consistent with a long and complex evolutionary history of the Cambarellinae. The group’s distribution has been modified extensively by geologic and climatic factors. Although there appear to be contrasting causes for cladogenesis between the two groups, they have similar diversification rates. In addition, these results showed that genital and morphological changes widely used in the subfamily in particular and in crayfish in general, should be compared with other kinds of evidence in order to make more robust use of morphological differences for evolutionary inferences.

## Supporting Information

Table S1 Voucher numbers and localities of the individuals analyzed. Proposed new taxa are indicated as *Cambarellus sp.*, and correspond to the terminal clades indicated with roman numerals in phylogeny (see Figure 3).
(DOCX)
