# Supplementary material for: Phylogeny and Evolutionary Patterns in the Dwarf Crayfish Subfamily (Decapoda: Cambarellinae)
Source: PLoS One. 2012 Nov 14;7(11):e48233. doi: 10.1371/journal.pone.0048233 (PMC3498282; doi:10.1371/journal.pone.0048233)
Supplement: Table S1 — Voucher numbers and localities of the individuals analyzed. Proposed new taxa are indicated as Cambarellus sp., and correspond to the terminal clades indicated with roman numerals in phylogeny (see Figure 3). (DOCX) [file pone.0048233.s001.docx]

**Table S1**. Voucher numbers and localities of the individuals analyzed. Proposed new taxa are indicated as *Cambarellus sp.*, and correspond to the terminal clades indicated with roman numerals in phylogeny (see Figure 3).

| **Point** | **Species id** | **Locality** | **Basin** | **Tissue/ Voucher** |
| --- | --- | --- | --- | --- |
| 1 | *Cambarellus blacki* | McDavid Landing cypress swamp, Escambia County, FL, USA | Escambia | KC4146, KC4147 |
| 2 | *Cambarellus diminutus** | 3 Mile W Grand Bay, US 90, Mobile County, AL, USA | Mobile | NMNH116911 |
| 3 | *Cambarellus lesliei** | Slough, 0.5 Mile S of Alabama Port, Mobile County,   junction Routes 163 And 188, AL, USA | Escatawpa | NMNH146621 |
| 4 | *Cambarellus ninae* | 6 Mile S, 1 mile W of Port Lavaca, Calhoun County, TX, USA | Guadalupe | NMNH133623 |
| 5 | *Cambarellus ninae*** | Rt. 1728 4.9 miles N. Rt. 35, Matagorda County, TX, USA | Colorado | DJ172 |
| 6 | *Cambarellus puer1233* | Ditch 0.3 mi from Navasota River, Brazos County, TX, USA | Brazos | BYUC 95-81 |
| 7 | *Cambarellus puer* | Drainage Ditch And Backwater of San Jacinto River,  Montgomery County, TX, USA | San Jacinto | NMNH220096 |
| 8 | *Cambarellus schmitti* | Branford, Small Spring, Suwannee County, FL, USA | Suwannee River | NMNH131694 |
| 9 | *Cambarellus schmitti* | NE. quadrant of junction of Interstate 10 and CR 279, Washington County, FL, USA | Choctahwachee | KC4215-KC4634 |
| 10 | *Cambarellus schmitti* | W. floodplain Choctawhatchee River at FL Hwy 2, Holmes County, FL, USA | Choctahwachee | KC6481 |
| 11 | *Cambarellus texanus* | Rt. 35 300 yds SW Rt. 1728, Matagorda County, TX, USA | Colorado | DJ146 |
| 12 | *Cambarellus texanus**** | Rt. 3013 @ Rt. 1093, Colorado Co., TX, USA | San Bernard | DJ173 |
| 13 | *Cambarellus texanus1210& 1211* | Roadside ditch on Co. Rd. 359 2.3 mi. N. of Fulshear, Fort Bend Co., TX, USA | Brazos | BYUC 95-70 |
| 14 | *Cambarellus shufeldtii* | Oxbow Lake Of The Red River, 0.6 Mile SW Of Boyd,  Lafayette County, AR, USA | Red | NMNH208635 |
| 15 | *Cambarellus shufeldtii* | Floodplain, 6.6 Mile N Of Gilmer, Upshur County, TX, USA | Sabine | NMNH219219 |
| 16 | *Cambarellus shufeldtii* | Bayou Dorcheat At US Highway 82,  Lafayette County, AR, USA | Red | NMNH208533 |
| 17 | *Cambarellus shufeldtii* | CR 184 at Quintette bridge, Santa Rosa County, FL, USA | Escambia |  |
| 18 | *Cambarellus shufeldtii* | Rt. 2879 at Little Cypress Creek, Harrison County, TX, USA | Sabine | DJ190 |
| 19 | *Cambarellus shufeldtii* | Rt. 79 at Mound Prairie Creek, Anderson County, TX, USA | Neches | DJ122 |
| 20 | *Cambarellus shufeldtii* | Unknown |  | BYUC 94-117 |
| 21 | *Cambarellus zempoalensis* | Ciénega Santa María, Michoacán, México | Cuitzeo |  |
| 22 | *Cambarellus zempoalensis* | Maravatío, Michoacán, México | Middle Lerma |  |
| 23 | *Cambarellus zempoalensis* | Zempoala Lagoons, Morelos, México | Interior |  |
| 24 | *Cambarellus zempoalensis* | Dren La Cinta, Michoacán, México | Middle Lerma |  |
| 25 | *Cambarellus zempoalensis* | Atécuaro, Michoacán, México | Cuitzeo |  |
| 26 | *Cambarellus zempoalensis* | Chiquimitío, Michoacán, México | Cuitzeo |  |
| 27 | *Cambarellus zempoalensis* | Aljojuca crater Lake, Puebla México | Interior |  |
| 28 | *Cambarellus zempoalensis* | Tepuxtepec dam, Edo. México, México | Middle Lerma |  |
| 29 | *Cambarellus zempoalensis* | Las Rosas, San Juan del Río, Querétaro, México | Pánuco |  |
| 30 | *Cambarellus zempoalensis* | Loma Alta, Michoacán, México | Cuitzeo |  |
| 31 | *Cambarellus patzcuarensis* | Lake Patzcuaro, in Janitzio island, Michoacan, México | Patzcuaro |  |
| 32 | *Cambarellus patzcuarensis* | Lake Zirahuén, at Opopeo Michoacan, México | Interior |  |
| 33 | *Cambarellus patzcuarensis* | Chapultepec spring, Michoacán, México | Patzcuaro |  |
| 34 | *Cambarellus patzcuarensis* | Spring at Tzurumutaro, Michoacán México | Pátzcuaro |  |
| 35 | *Cambarellus sp.* | La Mintzita spring, Michoacán, México | Cuitzeo |  |
| 36 | *Cambarellus chapalanus* | Cuerámaro dam, Tres Villas, Guanajuato, México | Turbio river |  |
| 37 | *Cambarellus chapalanus* | San Juanico Lake, Michoacán, México | Interior |  |
| 38 | *Cambarellus chapalanus* | Huaracha dam, Jalisco, México | Chapala |  |
| 39 | *Cambarellus chapalanus* | Zapotlán lake, Jalisco, México | Interior |  |
| 40 | *Cambarellus chapalanus* | San Julian, Jalisco, México | Verde/Santiago |  |
| 41 | *Cambarellus sp.* | San Miguel dam, Guanajuato, México | La Laja |  |
| 42 | *Cambarellus chapalanus* | San Isidro dam, Jalisco, México | Verde/Santiago |  |
| 43 | *Cambarellus prolixus* | Ajijic, at Chapala lake, Jalisco, México | Chapala |  |
| 44 | *Cambarellus chapalanus* | Camécuaro river, Michoacán, México | Chapala |  |
| 45 | *Cambarellus chapalanus* | La Luz spring, Michoacán, México | Chapala |  |
| 46 | *Cambarellus chapalanus* | Jamay, at Chapala Lake, México | Chapala |  |
| 47 | *Cambarellus chapalanus* | La Palma, Michoacán, México | Chapala |  |
| 48 | *Cambarellus prolixus* | Isla Patos, at Chapala Lake, México | Chapala |  |
| 49 | *Cambarellus prolixus* | Ocotlán, Jalisco, México | Chapala |  |
| 50 | *Cambarellus chapalanus* | Negritos, Michoacán, México | Chapala |  |
| 51 | *Cambarellus sp.* | Arroyo San Juan, Jalisco, México | Ameca river |  |
| 52 | *Cambarellus sp.* | Chapulimita stream, Jalisco, México | Ameca river |  |
| 53 | *Cambarellus sp.* | Los Veneros, Jalisco, México | Ameca river |  |
| 54 | *Cambarellus sp.* | Zacapu Lagoon, Michoacán, México | Angulo |  |
| 55 | *Cambarellus montezumae* | Quechulac crater Lake, Puebla, México | Interior |  |
| 56 | *Cambarellus montezumae* | Xochimilco, Estado de México, México | Valley of México |  |
| 57 | *Cambarellus sp.* | El Vegil dam, Amealco, Querétaro, México | Middle Lerma |  |
| 58 | *Cambarellus occidentalis* | Magdalena Lagoon, Jalisco, México | Interior |  |
| 59 | *Cambarellus occidentalis* | Tepic, Nayarit, México | Santiago |  |
|  | *Procambarus toltecae* | Huichihuayán, San Luis Potosí, México | Pánuco |  |
|  | *Procambarus acutus1* | Malila, Hidalgo, México | Pánuco |  |
|  | *Procambarus acutus2* | Canal Mante, Tamaulipas, México | Pánuco |  |
|  | *Procambarus llamasi1* | Motebello Lagoons, Chiapas, México | Grijalva-Usumacinta |  |
|  | *Procambarus llamasi2* | Guasimo, Tabasco, México | Grijalva-Usumacinta |  |
|  | *Procambarus clarkii* | Muzquiz, Coahuila, México | Salado |  |
|  | *Procambarus bouvieri* | Caltzontzin dam, Uruapan, Michoacán, México | Cupatitzio |  |
|  | *Orconectes deanae* | Route 717 at Norton Branch, Stephen Co., TX, USA. | Unknown | DJ42 |
|  | *Orconectes ronaldi* | Muddy Ck at Hwy 79, Logan Co., KY, USA. | Unknown | JC1424 |
|  | *Orconectes virilis1* | Buenaventura, Chihuahua, México | Santa María |  |
|  | *Orconectes virilis2* | Missouri, USA | Unknown | KC709 |
|  | *Cambarus brachydactylus^+^* | Humphreys Co., Blue Creek at bridge on SR 13, ~2mi S. of town of Waverly | Unknown | Genebank |
|  | *Cambarus maculatus* | Meramac River at Steelville Bridge, Crawford County, MO | Meramec | KC74 |
|  | *Cambarus pyronotus* | Ravine E. side SR 12 at Sweetwater, Liberty, FL, USA | Apalachicola | KC4941 |
|  | *Cambarus striatus* | Eglin Air Force Base, Alaqua creek N. of Bob Sikes RD, Walton County, FL, USA | Choctawhatchee | KC4941 |
|  | *Fallicambarus byersi* | County road 196 at Barrineau park, Escambia County, FL, USA | Perdido | KC4168 |
|  | *Fallicambarus caesius* | 5 miles E of Patmos, Hemptstead, AR, USA | Red River | JC2204 |
|  | *Fallicambarus fodiens* | West flood plain of Apalachacole river HWY 20, Bay County, FL, USA | Apalachicola | KC5238 |
|  |  |  |  |  |

*+* From the work on Buhay *et al*. 2007.
